# An evolutionary perspective on beautification: attractiveness, autonomy, and connectedness in men’s romantic judgment across mating contexts

**DOI:** 10.3389/fpsyg.2026.1898205

**Published:** 2026-07-31

**Authors:** Chun Zhao, Shaokang Chen, Hongya Zhou, Shiwen Qin, Yu Zou, Yimin Wang, Jien Guo, Xingyu Liu

**Affiliations:** 1 School of Fine Arts and Design, Xihua University, Chengdu, Sichuan, China; 2 School of the Arts, Universiti Sains Malaysia, Penang, Malaysia; 3 Faculty of Humanities and Arts, Macau University of Science and Technology, Macau, China; 4 School of Clothing Engineering and Design, Chengdu Qinggong Polytechnic University, Chengdu, Sichuan, China

**Keywords:** attractiveness, autonomy, beautification, connectedness, romantic judgment

## Abstract

**Introduction:**

Dress can be considered a visible and flexible form of female beautification that may communicate attractiveness as well as social and relational cues. However, less is known about how men interpret dress-based beautification cues when evaluating women as potential romantic partners across different mating contexts. The present study examined whether perceived attractiveness, autonomy, and connectedness derived from female dress jointly predict men’s romantic judgment and whether these associations differ between short-term and long-term relationship contexts.

**Methods:**

A between-subjects design was used in which heterosexual male participants (*N* = 405) were randomly assigned to either a short-term or long-term romantic evaluation condition. They rated ten female dress stimuli on attractiveness, autonomy, connectedness, and romantic judgment, yielding 4,050 observations. Data was analyzed using cross-classified linear mixed-effects models with all continuous predictors double-centered to remove between-participant and between-stimulus mean differences.

**Results:**

Relationship context moderated the associations with attractiveness and autonomy; attractiveness was more influential in short-term judgment, whereas autonomy showed a stronger association in long-term judgment. The interaction for connectedness was in the predicted direction but did not reach statistical significance.

**Conclusion:**

These findings suggest that dress-based cues are weighed differently across mating contexts. Attractiveness, autonomy, and connectedness converge to predict romantic judgments, with varying differences depending on the mating context.

## 1 Introduction

In the context of mating, physical appearance is one of the most immediate and consequential cues available to potential partners (Rhodes, 2006; Thornhill, 2003). Within seconds of encountering people, observers form impressions of attractiveness (Ritchie et al., 2017; Willis and Todorov, 2006), and these impressions shape subsequent preferences and behaviors, such as romantic judgments (Baxter et al., 2022; Eastwick and Finkel, 2008; Langlois et al., 2000; Selterman et al., 2015). Yet appearance is not a fixed property of the individual; it can be actively shaped and strategically projected. Beautification, the deliberate choices individuals make about dress or ornamentation, is one of the most accessible and widely practiced means of enhancing their attractiveness and managing the impressions they make on potential mates (Davis and Arnocky, 2022; Kowal et al., 2022). Such beautification choices are not merely expressions of aesthetic preference; they can also be understood as acts of self-presentation through which individuals signal desirability and position themselves favorably in the eyes of prospective partners (Ellison et al., 2006).

Critically, however, the significance of beautification is not exhausted by attractiveness alone. For men evaluating potential romantic partners, female dress-based beautification provides visual cues that can be perceived and assessed (Scheller et al., 2021). As beautification cues are available instantly (Willis and Todorov, 2006), they require no verbal exchange and can be interpreted even in the absence of direct interaction (Naumann et al., 2009), allowing men to form rapid trait-relevant impressions beyond physical attractiveness. Serving as highly visible and socially meaningful cues in person perception, dress can shape observers’ impressions of personality, status, social meaning, and attractiveness (Hester and Hehman, 2023; Johnson et al., 2014). Among the many social meanings that dress may communicate to potential partners, autonomy and connectedness are especially germane to mate evaluation: autonomy signals independence, self-expression, and self-directedness, while connectedness signals belonging, affiliation, and maintaining relationships (Blijlevens and Hekkert, 2019; Brewer, 1991; Fiske et al., 2007), both of which are qualities men assess when forming impressions of potential mates (Buss and Schmitt, 1993; Fletcher et al., 1999). Taken together, beautification can be conceptualized as both an attractiveness-enhancing strategy and a source of cues relevant to mate selection (Davis and Arnocky, 2022; Hester and Hehman, 2023).

Attractiveness, autonomy, and connectedness cues can be signaled by beautification; however, the evaluative weight assigned to each of these dimensions is unlikely to remain constant across all romantic contexts (Buss and Schmitt, 1993; Gangestad and Simpson, 2000). A man imagining a brief, immediate romantic relationship may rely on a different set of evaluative priorities than a man contemplating a sustained mutual investment in a long-term relationship (Li et al., 2002). The distinction between short-term and long-term relationship orientations represents distinct psychological frameworks, each imposing different demands on the male evaluator (Buss and Schmitt, 1993; Kenrick et al., 1993). These two relationship contexts are not merely different time horizons attached to the same judgment; they activate different sets of mate preferences, rendering different dimensions of beautification more or less relevant to partner evaluation (Buss and Schmitt, 2019; Kenrick et al., 1993). It is reasonable to expect that the evaluative significance of each dimension should not be uniform across relationship contexts but should vary with the different demands that each relational frame places on the male evaluator (Li et al., 2002).

Drawing on an evolutionary perspective, the present study examines how dress-based beautification cues, specifically perceived attractiveness, autonomy, and connectedness derived from female dress, jointly predict men’s romantic partner judgment and whether the relative influence of these three dimensions shifts in relationship context.

*RQ1*: Do attractiveness, autonomy, and connectedness perceived from female dress independently predict men’s romantic judgment?
*RQ2*: Does relationship context (short-term vs. long-term) moderate the effects of attractiveness, autonomy, and connectedness on men’s romantic judgment?

## 2 Literature review and hypotheses

### 2.1 Beautification in mating and romantic judgment

Beautification is broadly defined as the enhancement of appearance attractiveness through ornaments, modifications, and displays (Davis and Arnocky, 2022; Weiler et al., 2025). Evidence strengthens the idea that beautification behaviors are both widespread and systematically patterned. A large cross-cultural study spanning 93 countries found that such behaviors, including makeup use, hair grooming, clothing choices, and hygiene practices, were widespread, with most participants reporting spending more than 10 min per day on such activities (Kowal et al., 2022). The importance of beautification is further supported by evidence that a well-presented and attractive appearance can activate reward-related processes in observers’ brains, producing a “beauty premium” (Aharon et al., 2001; Cloutier et al., 2008) that translates into tangible advantages in real-world contexts (Langlois et al., 2000). Importantly, although both sexes engage in beautification, cross-cultural evidence suggests that women generally spend more time than men on such behaviors, indicating that female beautification is a particularly salient form of attractiveness presentation (Davis and Arnocky, 2022; Kowal et al., 2022).

A growing quantity of research situates female appearance attractiveness enhancement within an evolutionary framework, examining it as a phenomenon fundamentally linked to mating competition. From Paleolithic body ornamentation (Gilligan et al., 2024) to ancient Egyptian cosmetic practices (Scott, 2016), the beautification pattern can be found across history (Wang et al., 2021). The cross-cultural ubiquity of beautification, combined with the substantial time, effort, and financial resources individuals invest in it, suggests that these behaviors may reflect evolved dispositions that transcend any single cultural context (Arnocky et al., 2023; Davis and Arnocky, 2022). Sexual selection theory provides the foundational evolutionary framework for understanding female beautification as mating-relevant behavior, favoring traits and behaviors that enhance mate acquisition even when they are costly or only indirectly related to survival (Davis and Arnocky, 2022; Puts, 2016). In line with this perspective, it is reasonable to describe female appearance attractiveness enhancement as a self-promotion strategy that can increase reproductive success by rendering oneself more attractive than rivals and, in turn, increasing one’s mate value (Davis and Arnocky, 2022). This interpretation situates it within broader processes of mate attraction and reproductive competition; female beautification is best understood not merely as attractiveness enhancement but as a potentially adaptive form of mating effort directed toward male evaluators.

Clothing occupies a particularly central role in increasing attractiveness among the various forms of female beautification. Unlike more fixed appearance attributes such as facial morphology or body shape, clothing is rapidly alterable and highly visible, making it an especially flexible cue through which women can enhance attractiveness and communicate mating intentions (Elliot and Pazda, 2012; Grammer et al., 2004; Hester and Hehman, 2023; Pazda et al., 2012). Because clothing can be deliberately adjusted in style, fit, color, and degree of body display, it provides a particularly immediate means of shaping perceived attractiveness and influencing observers’ impressions of the wearer. In this sense, female dress may serve not merely as decoration but as a strategic beautification cue that contributes to mate-relevant evaluation and competitive attractiveness (Gouda-Vossos et al., 2019). Evidence from studies of women’s ornamentation and dress behavior further supports the idea that clothing-based beautification is closely tied to reproductive and mating-related motives. For example, Haselton et al. (2007) reported that self-grooming and ornamentation through attractive dress increased during the fertile phase of the ovulatory cycle, suggesting that attractiveness covaries with periods of heightened mating relevance. Similarly, Durante et al. (2008) found that women preferred sexier and more revealing clothing at high fertility, and subsequent work by Durante et al. (2011) interpreted such shifts partly in terms of intensified female–female competition. As Davis and Arnocky (2022) noted in their review, beautification practices also vary predictably with mating-related factors, which are consistent with an evolved, facultatively expressed behavioral repertoire. Taken together, these findings suggest female beautification that enhances perceived attractiveness is an evolutionarily shaped, strategically responsive behavior that tracks reproductive conditions and is directed, at least in part, toward the evaluative judgments of male observers. Hypothesis 1 is proposed as follows:

*H1*: The attractiveness perceived from female dress will positively predict men’s romantic judgment.

Critically, male observers are the primary recipients of female beautification cues (Buss, 1989), so the impressions men form from female appearance choices carry consequences for romantic judgment. Understanding how such judgments are understood, therefore, requires examining female dress as a source of perceived attractiveness while also considering the broader social and relational qualities male observers infer from such choices in mate evaluation.

### 2.2 Female beautification as cues of autonomy and connectedness

If female beautification serves multiple functions, its influence on mate evaluation cannot be reduced to attractiveness enhancement alone. Humans have evolved specialized cognitive mechanisms for making rapid inferences from limited visible cues, and these inferences guide subsequent social and mating behaviors (Buss, 1989; Cosmides and Tooby, 2013). Dress is one particularly salient class of such cues; unlike fixed physical features, clothing and adornment are intentionally constructed and culturally coded, making them especially diagnostic signals of a wearer’s personality, status, and social identity (Hester and Hehman, 2023; Johnson et al., 2014). Female dress-based beautification, therefore, conveys a range of interpersonal and social meanings that may shape how observers evaluate potential mates beyond attractiveness alone.

Among the many social meanings that dress may communicate to potential partners, autonomy and connectedness are especially germane to mate evaluation, both of which are qualities men assess when forming impressions of potential mates (Blijlevens and Hekkert, 2019; Brewer, 1991; Buss and Schmitt, 2019; Cialdini et al., 1999; Fiske et al., 2007; Fletcher et al., 1999; Markus and Kitayama, 1991; Reed, 2004). In the present study, connectedness refers to the extent to which a female target is perceived as socially attuned, relationally oriented, and aligned with others, whereas autonomy refers to the extent to which a female target is perceived as self-directed, independent, and agentic (Blijlevens and Hekkert, 2019; Brewer, 1991; Fiske et al., 2007). Normative dress choices, such as those that align with mainstream fashion or social conventions, may signal to male observers that a woman prioritizes social belonging and relational attunement, thereby cueing perceptions of connectedness. Conversely, dress choices that foreground personal style, distinctiveness, or unconventionality may cue perceptions of autonomy by conveying that the woman prioritizes self-expression and individual agency over social conformity.

The evolutionary basis for why each dimension carries evaluative relevance in romantic judgment can be traced to distinct adaptive pressures. On one hand, connectedness, belonging to a cooperative social group, confers substantial fitness benefits, including resource pooling, collective protection, and enhanced reproductive opportunities that solitary individuals cannot achieve (Axelrod and Hamilton, 1981; Cosmides and Tooby, 2013). These ancestral pressures may have shaped a perceptual sensitivity to cues signaling group membership and social alignment; individuals whose appearance communicates belonging and cooperative disposition are therefore perceived as offering the relational benefits associated with group cohesion (Berghman and Hekkert, 2017; Blijlevens and Hekkert, 2019). On the other hand, within the safe confines of the group, we benefit from standing out to some extent; adaptation within a cooperative group also favors individuals capable of individual differentiation and resource competition (Berghman and Hekkert, 2017; Blijlevens and Hekkert, 2019). Group members must simultaneously cooperate and compete for mates, status, and resources, which creates selection pressure for autonomy toward the display of personal competence and social distinctiveness. Thus, we value autonomy from our reference group.

Empirical evidence is consistent with this broader account. Regarding autonomy, research has shown that individuals who behave independently of group norms are evaluated more favorably as potential romantic partners, especially when men evaluate women (Griskevicius et al., 2006; Hornsey et al., 2015). For connectedness, research on partner preferences shows that men and women reliably value agreeableness and relational orientation, supportive qualities in potential romantic partners (Fletcher et al., 1999; Sprecher and Regan, 2002; Valentine et al., 2020). These two dimensions, however, should not be treated as opposing tendencies that mutually exclude one another. Rather, they represent distinct axes of social inference to different contexts or forms, along which a given appearance may carry meaningful cues of value independently and simultaneously (Blijlevens and Hekkert, 2019; Hester and Hehman, 2023). Female dress choices are better understood as expressions reflecting the degree to which a woman’s self-presentation emphasizes individual differentiation relative to social alignment, rather than as categorical cues of either quality alone. Research on optimal distinctiveness suggests that individuals do not maximize either conformity or individuality but seek a contextually calibrated balance between the two and that excessive uniformity of appearance motivates compensatory differentiation as a means of restoring a sense of self (Brewer, 1991; Hornsey et al., 2015). Hypotheses 2 and 3 are proposed as follows:

*H2*: The connectedness perceived from female dress will positively predict men’s romantic partner judgment.

*H3*: The autonomy perceived from female dress will positively predict men’s romantic partner judgment.

The evaluative significance of these cues for male observers, however, is unlikely to be uniform across all romantic contexts. The extent to which autonomy or connectedness cues derived from female dress influence men’s romantic judgment may depend on the nature of the relationship under consideration, a possibility examined in the following section.

### 2.3 Relationship context as a moderator of beautification-based romantic judgment

Although females’ beautification choices may shape multiple interpersonal impressions in male observers, the evaluative significance of these impressions is unlikely to be constant across romantic contexts. From an evolutionary perspective, short-term and long-term mating represent distinct adaptive strategies, each associated with different goals, relational constraints, and informational priorities (Buss and Schmitt, 1993; Gangestad and Simpson, 2000). In the present study, short-term judgment refers to men’s evaluation of a female target within a short, flexible, and casual relationship evaluation context, whereas long-term judgment refers to evaluation within a long, committed, and serious relationship. Although these two forms of judgment are not mutually exclusive in real-world romantic experience, they represent meaningfully distinct evaluative orientations (Kenrick et al., 1990; Li and Kenrick, 2006; Regan et al., 2000). A framework that treats men’s romantic judgment as uniform across contexts is therefore likely to obscure meaningful variation in how appearance-based cues are weighed and interpreted.

Sexual strategies theory holds that short-term and long-term mating contexts can activate systematically different preference criteria and produce different patterns of mate assessment in men (Buss and Schmitt, 2019; Li and Kenrick, 2006). Consistent with this context-dependent view, Liang et al. (2024) found that men’s evaluations of female beautification strategies involved not only attractiveness but also perceived loyalty in long-term mating evaluation. This distinction has direct implications for how appearance-based cues that male observers derive from female dress choices, specifically perceived attractiveness, autonomy, and connectedness, are expected to function as evaluative inputs across the two mating contexts (Davis and Arnocky, 2022; Fletcher et al., 1999; Regan et al., 2000). Each of these cues carries a distinct set of interpersonal meanings, and the relevance of those meanings to male romantic judgment is expected to vary with the relational frame in which evaluation occurs.

Appearance attractiveness represents the most immediately processed cue available to a male observer, and its relevance to mate evaluation is well established. Evolutionarily, physical attractiveness has been theorized as a reliable indicator of reproductive value, hormonal health, and genetic quality (Gangestad and Scheyd, 2005; Rhodes, 2006; Thornhill and Gangestad, 1999), making it a high validity cue of mate quality. Dress choices constitute a particularly consequential medium through which attractiveness is communicated, as clothing can accentuate or reveal bodily features associated with reproductive value while simultaneously concealing features that deviate from idealized standards, rendering dress a flexible instrument for beautification of female attractiveness (Prokop et al., 2026; Sidhu et al., 2021). Accordingly, attractiveness derived from female dress is expected to exert a particularly strong influence in short-term mating contexts, where the evaluative horizon is narrow, and the adaptive premium on efficient assessment is high; in long-term contexts, attractiveness may remain relevant, but its relative weight is expected to diminish as other partner qualities bearing on sustained relational functioning become more consequential (Gangestad and Simpson, 2000; Li and Kenrick, 2006).

The role of perceived autonomy communicated through female dress may vary in context. Autonomy cues in dress choices imply independence, such as personal agency or creative confidence, which are qualities that may enhance perceived desirability in contexts where individuality and self-sufficiency are valued (Blijlevens and Hekkert, 2019; Griskevicius et al., 2006). Such autonomy or independence, while not inherently undesirable, may invite male observers to infer lower relational embeddedness or reduced orientation toward long-term relationships and thus be weighted less favorably in long-term mating contexts (Buss and Schmitt, 2019; Lopes et al., 2017). Whereas in short-term mating contexts, autonomy cues carry less evaluative cost, as the relational demands of such contexts do not foreground partner reliability or long-term cooperative investment as primary criteria (Kenrick et al., 1993). The evaluative weight assigned to autonomy cues by male observers, therefore, may be less in the long term than in the short term, not because independence is valued intrinsically in the latter, but because the relational demands of the former foreground different inferential priorities.

A parallel but inverse pattern may characterize the evaluative role of connectedness cues. Beautification choices that convey social belonging and group attunement imply cooperativeness, relational orientation, and a disposition toward norms; such qualities are broadly associated with partner suitability in contexts requiring sustained interpersonal investment (Fletcher et al., 1999; Valentine et al., 2020). Parental investment theory suggests that because long-term relationships involve substantially greater investment, male evaluators in such contexts become particularly sensitive to cues indicating a partner’s cooperative disposition and capacity for relational commitment (Buss and Schmitt, 2019; Kenrick et al., 1993). Consistent with this, empirical evidence indicates that men allocate significantly greater evaluative weight to kindness and warmth when assessing long-term relative to short-term partners (Li et al., 2002; Li and Kenrick, 2006), and that ideal partner standards for long-term relationships show a stronger loading on warmth-trustworthiness dimensions compared to short-term contexts (Fletcher et al., 1999). Beautification cues that communicate connectedness may be associated with indicators for these relevant qualities, acquiring heightened evaluative significance in long-term judgment. In short-term contexts, by contrast, the interpersonal qualities implied by connectedness may carry less immediate relevance, as evaluative priority shifts toward more proximate indicators of physical attractiveness rather than longer-term relational compatibility.

Taken together, these considerations suggest that the predictive roles of attractiveness, autonomy, and connectedness derived from females’ beautification choices in shaping men’s romantic evaluation are unlikely to be identical across these two contexts. Appearance attractiveness may exert a stronger and more consistent influence on men’s short-term desirability judgments, while autonomy and connectedness cues may carry differential and potentially context-dependent implications depending on the relational frame adopted by the male observer. The present study examines these possibilities directly, investigating whether and how attractiveness, autonomy, and connectedness derived from female dress choices differentially predict men’s romantic judgment across short-term and long-term mating contexts. The present study further proposed the following hypotheses:

*H4*: Relationship context will moderate the relationship between perceived attractiveness and romantic judgment, such that attractiveness will have a stronger positive effect in the short-term context than in the long-term context.

*H5*: Relationship context will moderate the relationship between perceived autonomy and romantic judgment, such that autonomy will have a weaker positive effect in the long-term context than in the short-term context.

*H6*: Relationship context will moderate the relationship between perceived connectedness and romantic judgment, such that connectedness will have a stronger positive effect in the long-term context than in the short-term context.

## 3 Methods

This study examined whether perceived attractiveness, autonomy, and connectedness derived from beautification-related appearance cues predict romantic judgment, and whether these predictive effects differ across short-term and long-term relationship contexts. A between-subjects design was adopted for the relationship context, such that participants were assigned to either a short-term judgment condition or a long-term judgment condition. In both conditions, participants viewed the same set of beautification-related stimuli and rated each target on perceived attractiveness, perceived autonomy, perceived connectedness, and romantic judgment. This design allowed the study to test whether the cue-based predictors of romantic evaluation vary as a function of relationship context. The following sections detail the stimuli, participants, instruments, data collection procedures, and data analysis.

Ethical approval was granted by the Institutional Ethical Review Board. Participants were fully briefed on the study’s purpose, and all data was anonymized. They received small compensation for their involvement.

### 3.1 Stimuli selection

The stimuli consisted of a final set of 10 female dress images selected through a three-stage procedure: (a) creating an initial pool of candidate stimuli, (b) obtaining ratings of perceived connectedness, autonomy, and attractiveness from an independent sample of non-design participants, and (c) selecting the final stimuli based on their distribution across the three theoretically relevant perceptual dimensions. Because the present study aimed to isolate dress-based beautification cues rather than evaluate real women as whole-person targets, standardized dress images were used instead of photographs of actual wearers. This stimulus-control strategy excludes potential confounds from facial attractiveness, body shape, posture, age, expression, skin exposure, and contextual background, although it also limits the ecological richness of the stimuli. This procedure was adopted to ensure that the final stimuli represented a meaningful range of beautification-related appearance cues.

An initial pool of 60 female dress images was digitally modeled by the authors specifically for the present study. The stimuli were created through a digital modeling process in which garment forms were constructed by the authors and subsequently refined using AI-assisted image editing tools to ensure consistency (such as presentation clarity and background uniformity) across stimuli. The experimental stimuli themselves were author-modeled and AI-assisted in their refinement. The stimuli were created to represent varied levels of perceived attractiveness, autonomy, and connectedness while maintaining a consistent visual format across images. Because the present study aimed to test dress-based beautification cues, all images were standardized so that participants’ judgments were based primarily on the dress rather than on facial attractiveness, branding, or contextual information. Images depicting identifiable logos, brand labels, or culturally distinctive attire were excluded to minimize confounding variation unrelated to the core beautification cues of interest. The remaining images were standardized in viewing angle, resolution, aspect ratio, and background color to reduce incidental variation in image quality and presentation.

The initial pool of 60 images was rated by an independent sample of non-design participants who did not participate in the main study (*N* = 30). Participants rated each image on perceived attractiveness, autonomy, and connectedness using 7-point Likert scales. These pilot ratings were used only for stimulus selection and were not included in the main analyses.

Because the present study examined beautification as a positive appearance-based cue, the final stimuli were required to show at least moderate endorsement on one of the three theoretically relevant dimensions. Specifically, each selected dress image had to receive a mean score above the scale midpoint on at least one dimension, attractiveness, autonomy, or connectedness. This criterion ensured that every stimulus carried a discernible beautification-related cue. At the same time, the stimuli were not required to score highly on all three dimensions, allowing the final set to preserve meaningful variation in the relative strength of attractiveness, autonomy, and connectedness cues.

**Figure 1:**
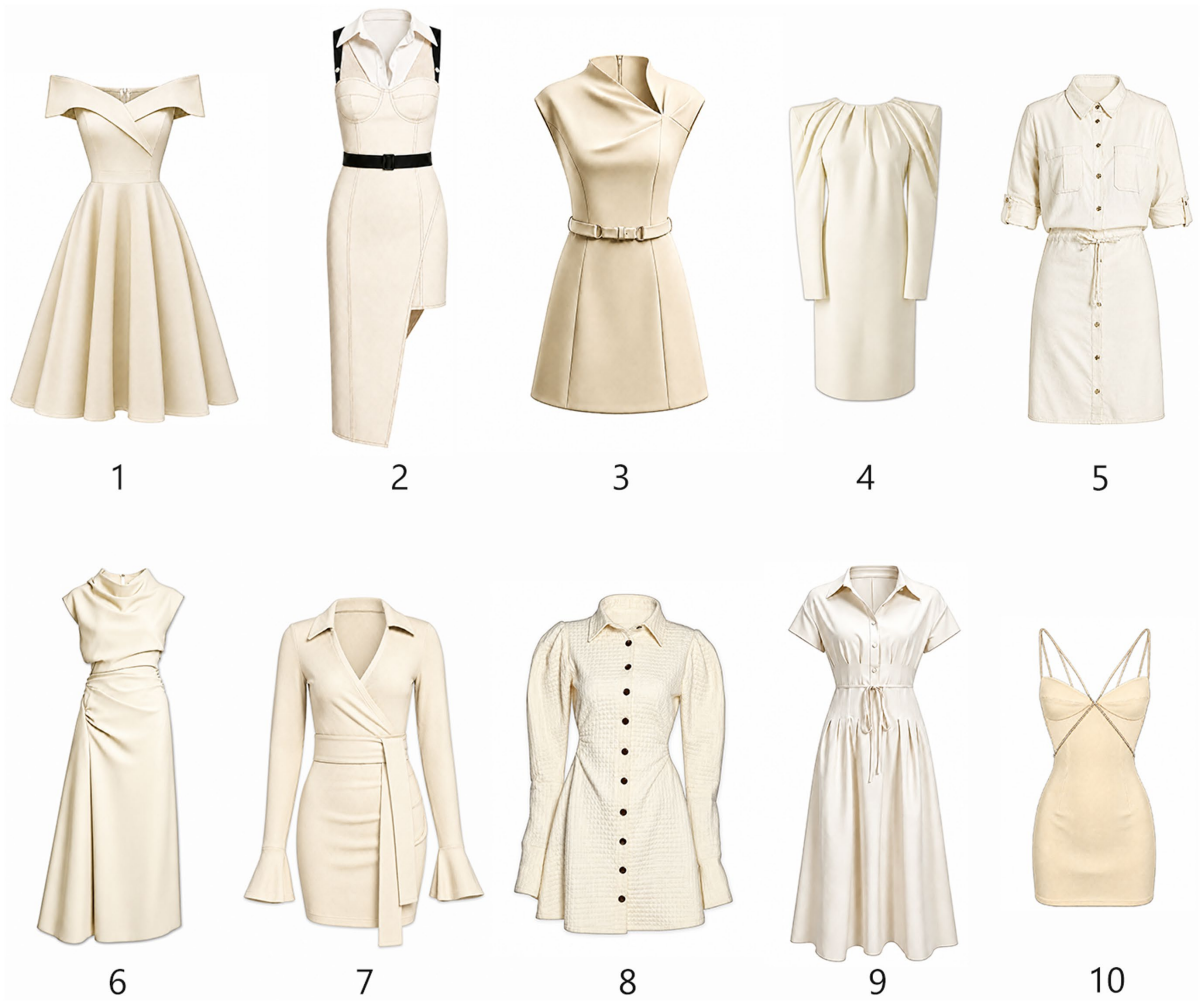
The final set of stimuli.

A total of 10 dress stimuli were selected for the formal study. Based on the independent pilot ratings, the selected stimuli showed mean attractiveness scores ranging from 3.5 to 6.1, mean connectedness scores ranging from 3.2 to 6.2, and mean autonomy scores ranging from 3.8 to 6.5. Figure 1 presents the final set of 10 dress stimuli.

### 3.2 Participants

Heterosexual male perceivers were targeted exclusively because the study concerned men’s evaluation of female beautification cues in the context of opposite-sex romantic judgment. Only individuals who self-identified as male and reported heterosexual orientation were eligible to participate. Additional inclusion criteria required participants to be aged 18 to 45 years. Participants were required to complete the survey in a single uninterrupted session; those who failed an attention check item embedded within the survey were excluded from analysis.

Of the 466 participants in the recruitment, valid data was collected from 405 subjects, with 61 excluded due to inaccuracies: (a). failed an attention check item; (b). uncompleted questionnaire; (c). the entire questionnaire contained ≥ 6 consecutive questions with the same score; and (d). alternating extreme responses: the number of adjacent transitions with |*Δ*| ≥ 5 exceeded 6 across the questionnaire. A total of 405 heterosexual men completed the study (*M* = 24.12 years, SD = 2.37; age range: 20–36 years). Participants were recruited from a comprehensive university in a metropolis in southwest China through convenience sampling facilitated by the research team’s institutional affiliation. The city is a major metropolitan center in southwestern China with a large and diverse university population, making it practically feasible to recruit participants for the present study. Participants were randomly assigned to either the short-term evaluation condition (*N* = 203) or the long-term evaluation condition (*N* = 202). The final analytic sample yielded 4,050 observations (405 participants × 10 dress stimuli) for the formal analyses.

A sample power analysis conducted in G*Power (Faul et al., 2007) indicated that a sample of *N* = 405, at *α* = 0.05 and power = 0.80, was sufficient to detect a small-to-medium fixed effect (f^2^ ≥ 0.04) in a multilevel regression model. Ethical approval was granted by the Institutional Ethical Review Board before data collection. All participants provided informed consent before beginning the study and were debriefed upon completion. Participants received financial compensation commensurate with the estimated completion time.

### 3.3 Instruments

Four measures were administered for each of the 10 dress stimuli: perceived attractiveness, perceived autonomy, perceived connectedness, and romantic judgment. All items were rated on a 7-point Likert scale ranging from 1 (not at all) to 7 (very much), with higher scores indicating greater endorsement of the construct in question.

The predictor measures operated at complementary but conceptually distinct levels of perception, consistent with the theoretical framework proposed by Blijlevens and Hekkert (2019). Following Blijlevens et al.’s (2017) Aesthetic Pleasure in Design Scale, perceived attractiveness was assessed as an aesthetic property of the dress itself, capturing the direct response to the garment as a visual object. Perceived autonomy, connectedness, and romantic judgment, by contrast, were assessed as social inferences or further judgment about the hypothetical wearer, capturing the interpersonal meanings and self-determination that observers derive from dress-based cues, following Blijlevens and Hekkert (2019), who conceptualized these dimensions as meanings that observers derive from the wearer rather than properties of the object itself. This distinction reflects the dual nature of dress, as the dress simultaneously carries aesthetic properties that are directly perceivable and communicates information about the wearer that observers actively infer (Blijlevens and Hekkert, 2019). The present study adopts this framework, treating predictors as operating at different perceptual levels that together constitute the full evaluative response to dress-based beautification cues. Importantly, all constructs remain perceptual responses derived from viewing the same dress stimulus, which is what the present study examines.

Perceived attractiveness was assessed with three items adapted from established measures of appearance-based attractiveness, the Blijlevens et al. (2017) pleasing-to-see scale. This scale has demonstrated high reliability and cross-study adaptability across diverse stimulus sets and participant samples (Chen et al., 2025; Ma et al., 2025; Suhaimi et al., 2023). Items asked participants to rate the extent to which each dress appeared physically attractive, appealing, and good-looking. Attractiveness was assessed using three statements: (a) “This is a beautiful dress.,” (b) “This is an attractive dress.,” and (c) “This dress is pleasing to see.” Internal consistency in the present sample was *α* = 0.867.

Perceived autonomy and connectedness were assessed with three items for each, adapted from Blijlevens and Hekkert’s (2019) scale designed to capture the extent to which the wearer of each dress appeared self-directed, independent, and distinctive or appeared socially attuned, relationally oriented, and aligned with others. In autonomy, three items assessed evaluation: (a) “This dress signals that the wearer values personal independence and self-expression.,” (b) “The wearer of this dress appears to enjoy being unique and different from others.,” and (c) “The wearer of this dress seems to prioritize their own individuality.” Items for connectedness measurement including: (a) “This dress signals that the wearer values closeness and belonging with others.,” (b) “The wearer of this dress appears to prioritize harmony and fitting in with others.,” and (c) “The wearer of this dress seems to prioritize their relationships.” Internal consistency for autonomy was α = 0.856, and for connectedness was *α* = 0.895.

Romantic judgment was assessed using items adapted from short-term and long-term mate desirability measures used in evolutionary mate-preference research (Buss and Schmitt, 1993; Dragostinov and Booth, 2025; Haselton and Miller, 2006). These measures explicitly distinguish romantic evaluation across short-term and long-term relational contexts by asking participants to evaluate the desirability of a target as either a short-term or long-term partner. In the present study, participants evaluated each dress stimulus under only one assigned relational condition. Those assigned to the short-term condition completed the short-term romantic judgment items, whereas those assigned to the long-term condition completed the parallel long-term items. In the short-term condition, three items assessed short-term romantic evaluation: (a) “How desirable would you find the wearer of this dress as a short-term dating partner?,” (b) “How much would you like to date the wearer of this dress briefly?,” and (c) “How romantically attractive is the wearer of this dress for a casual relationship?” Parallel items were administered in the long-term condition: (a) “How desirable would you find the wearer of this dress as a long-term dating partner?,” (b) “How much would you like to be in a committed relationship with the wearer of this dress?,” and (c) “How romantically attractive is the wearer of this dress for a serious relationship?” All items were rated on a 7-point scale ranging from 1 (not at all) to 7 (very much), with higher scores indicating stronger romantic evaluation within the corresponding relational context. Internal consistency for the short-term was *α* = 0.757, and for the long-term was *α* = 0.764.

Overall, internal consistency was acceptable to excellent across the four measures, with Cronbach’s alpha values ranging from 0.757 to 0.895 (Cronbach, 1951). The overall KMO value was 0.811, which is classified as meritorious (Kaiser, 1974), indicating that the intercorrelation pattern is appropriate for factor analysis. Bartlett’s test of sphericity was statistically significant (*p* < 0.01).

### 3.4 Data collection procedure

Data was collected via an online survey platform. Participants were presented with an information sheet describing the general purpose of the research and their rights as participants. After providing electronic informed consent, participants completed a brief demographic questionnaire assessing age, self-identified gender, and sexual orientation.

Participants were then randomly assigned by the survey platform to either the short-term or long-term relationship evaluation condition. Following the assignment, participants read a brief condition-specific prompt instructing them to imagine evaluating the dress images from the perspective of either a short-term or long-term romantic relationship. In the short-term condition, participants were instructed to imagine evaluating the wearer of each dress as a potential partner for a brief, casual romantic relationship. In the long-term condition, participants were instructed to imagine evaluating the wearer of each dress as a potential partner for a committed and serious romantic relationship. This contextual framing has been used in prior mate evaluation research to reliably activate condition-appropriate evaluative orientations (Buss and Schmitt, 1993; Kenrick et al., 1993).

The 10 dress stimuli were presented sequentially in a randomized order for each participant. For each image, participants rated the dress on the four measures described in Section 3.3 (perceived attractiveness, perceived autonomy, perceived connectedness, and romantic judgment) before proceeding to the next image. To ensure that responses reflected independent evaluations, participants were not permitted to return to previously rated images. An attention check item (“Please select the number 4 for this question”) was embedded at the midpoint of the survey; participants who responded incorrectly were excluded from analysis.

To ensure data consistency and reliability, participants were required to complete the survey without interruption. This approach helped ensure that participants maintained a clear memory of the images, thus improving the accuracy of their responses. The mean completion time was approximately 18–25 min. Upon completion, participants were shown a debriefing screen describing the study’s objectives and thanked for their participation. All data were anonymized before analysis.

### 3.5 Data analysis

The confirmatory factor analysis (CFA) was conducted before the formal analyses to assess discriminant validity among the three predictors. As the data had a cross-classified structure with each participant rating multiple dress stimuli and each dress stimulus being rated by multiple participants, ordinary least squares regression would violate the assumption of independent observations. Accordingly, cross-classified linear mixed models were estimated using maximum likelihood (ML) estimation, implemented in the lme4 package (Bates et al., 2015), and statistical tests for fixed effects were conducted using Satterthwaite degrees of freedom as implemented in the lmerTest package. All continuous predictors were double-centred by residualizing each predictor on both dress identity and participant identity. This approach reduces the possibility that the observed associations are merely attributable to some dresses being rated generally higher across all dimensions, some participants giving consistently higher ratings, or uncontrolled visual characteristics of specific dress stimuli simultaneously elevating multiple predictors and outcome ratings. Random intercepts were specified for both participants and dress stimuli, and random slopes for the three double-centred predictors were specified over participants, to account for non-independence within each clustering unit.

A null model containing only random intercepts was first estimated to partition the total variance and establish baseline model fit. The intraclass correlation coefficient (ICC) was computed from this model to quantify the proportion of variance in romantic judgment attributable to systematic differences between participants and between stimuli.

To address RQ1 and test H1 to H3, a fixed-effects model (Model 1) was estimated with perceived attractiveness, autonomy, and connectedness as simultaneous double-centered predictors of romantic judgment. This model tested whether perceived attractiveness, autonomy, and connectedness, each positively predicted men’s romantic judgment, corresponding to H1, H2, and H3. Cross-classified random intercepts for participants and dress stimuli were included to account for the non-independence of observations, along with random slopes for all three predictors varying by participant, allowing each man’s sensitivity to attractiveness, autonomy, and connectedness to differ. Model fit relative to the null model was evaluated using a likelihood ratio test (Δχ^2^).

To address RQ2 and test H4 to H6, a second model (Model 2) was estimated that added the binary relationship context variable (0 = short-term, 1 = long-term) as a main effect and its two-way interactions with each of the three predictors, corresponding to H4, H5, and H6. The random effects structure was retained from Model 1. The significance of each interaction term was assessed using Wald t-tests. Simple slopes for each predictor were computed separately within each relationship context (short-term and long-term) to characterize the nature of significant interactions. Model fit improvement relative to Model 1 was again evaluated using a likelihood ratio test. Marginal *R*^2^ (variance explained by fixed effects alone) was computed following the approach of Nakagawa and Schielzeth (2013). The significance threshold was set at *α* = 0.05 for all tests.

## 4 Results

The reliability and validity of all scales were evaluated before data analysis. Reliability was assessed using Cronbach’s alpha (α), and validity was examined using the Kaiser-Meyer-Olkin (KMO) measure of sampling adequacy alongside Bartlett’s test of sphericity. Internal consistency was acceptable to excellent across all four scales. The overall KMO value was 0.811, and Bartlett’s test of sphericity was statistically significant (*p* < 0.01).

**Figure 2:**
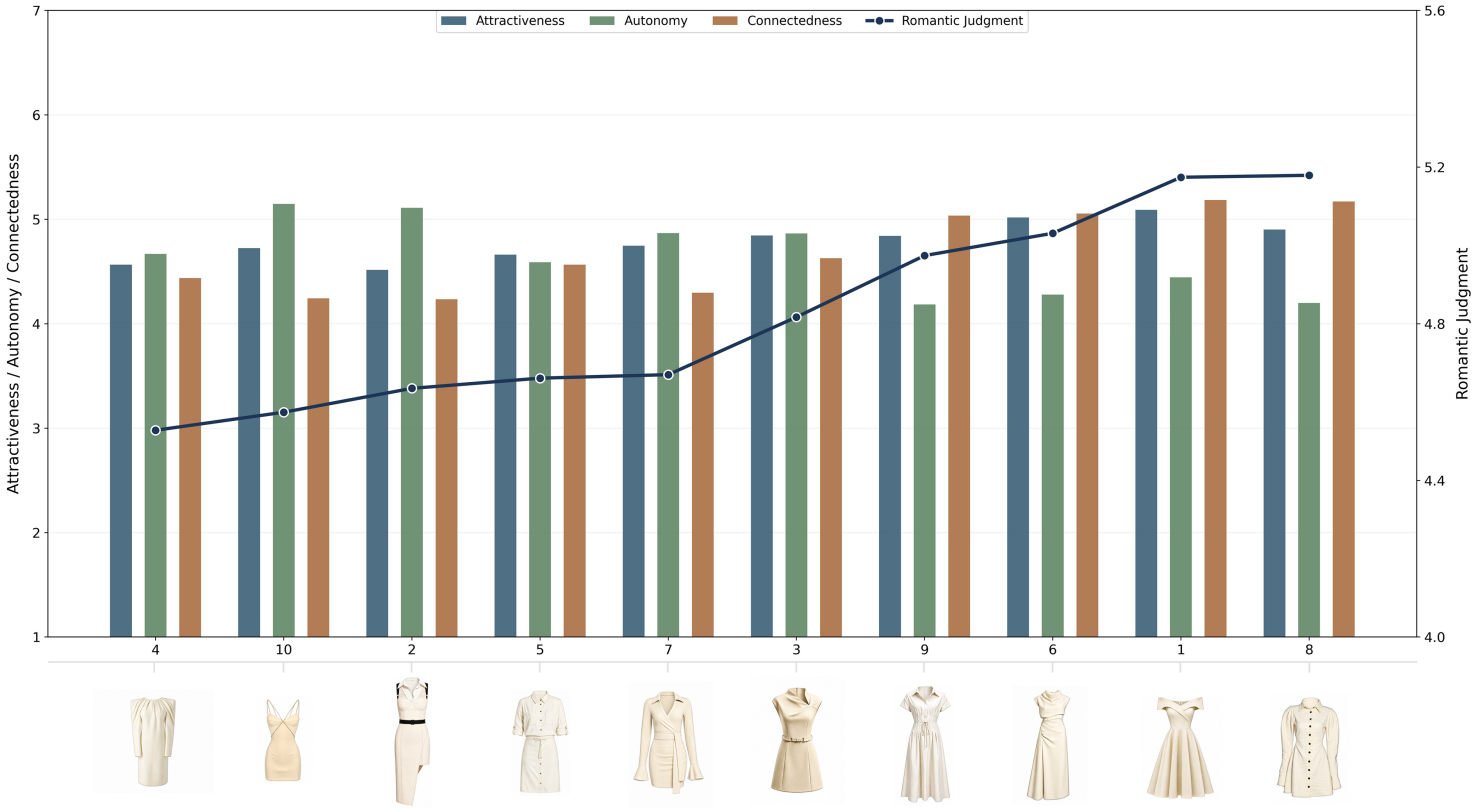
Dress-level mean ratings of attractiveness, autonomy, connectedness, and romantic judgment.

The results of the participants’ ratings of the scales were summarized, including the mean scores of attractiveness, autonomy, connectedness, and romantic judgment for each dress, as shown in Table 1 and Figure 2.

**Table 1:** Dress-level descriptive statistics for connectedness, autonomy, attractiveness, and romantic judgment (overall).

Dress	Connectedness		Autonomy		Attractiveness		Romantic judgment	
	*M*	SD	*M*	SD	*M*	SD	*M*	SD
1	5.184	1.141	4.444	1.410	5.091	1.009	5.174	0.850
2	4.235	1.005	5.109	0.842	4.515	0.811	4.635	0.838
3	4.628	0.817	4.865	0.945	4.844	0.884	4.817	0.780
4	4.438	0.882	4.669	0.932	4.565	0.786	4.528	0.714
5	4.566	0.800	4.589	0.990	4.662	0.930	4.661	0.781
6	5.055	0.916	4.278	1.048	5.017	0.812	5.031	0.762
7	4.296	0.988	4.868	0.938	4.746	0.771	4.670	0.674
8	5.170	1.162	4.198	1.299	4.901	1.560	5.179	1.004
9	5.035	1.131	4.184	1.047	4.842	1.261	4.974	0.907
10	4.243	0.896	5.147	1.236	4.723	0.826	4.574	0.761

Mean and SD are calculated for each dress using composite variables.

As shown in Figure 2, the ten dress stimuli were ordered from lowest to highest romantic judgment. Visual inspection indicated a gradual increase in romantic judgment across the stimulus sequence, from approximately 4.5 for the lowest-ranked stimuli to above 5.0 for the highest-ranked stimuli. In particular, the highest-ranked dress stimuli tended to show middle-to-high attractiveness and connectedness scores while still preserving middle autonomy. Several stimuli with relatively high autonomy, such as stimuli 10 and 2, did not receive high romantic judgment scores, whereas some of the most preferred stimuli showed only moderate autonomy.

The CFA was conducted before the main analyses to evaluate the discriminant validity of the three perceived dress cue dimensions. A three-factor model specifying attractiveness, autonomy, and connectedness as distinct latent factors was compared against a one-factor model in which all items loaded onto a single common factor. The three-factor model demonstrated acceptable fit, *χ*^2^ (24) = 307.84, *p* < 0.001, CFI = 0.986, RMSEA = 0.054, and SRMR = 0.026. The one-factor model fit the data substantially worse, *χ*^2^ (27) = 13811.23, *p* < 0.001, CFI = 0.329, RMSEA = 0.355, and SRMR = 0.231. A scaled chi-square difference test confirmed that the three-factor model fit significantly better than the one-factor alternative, ΔChisq (3) = 26,271, *p* < 0.001. Inter-factor correlations among the three latent factors were low (attractiveness with autonomy: *r* = 0.051; attractiveness with connectedness: *r* = 0.205; autonomy with connectedness: *r* = 0.056), all well below the 0.85 threshold commonly used to identify discriminant validity concerns. These results support treating attractiveness, autonomy, and connectedness as empirically distinct constructs in subsequent analyses.

A null model containing only random intercepts was estimated to partition variance and establish baseline fit. The intraclass correlation coefficient (ICC) was 0.165, indicating that approximately 16.5% of the variance in romantic judgment was attributable to systematic differences between participants and dress stimuli. This clustering justified the use of cross-classified mixed models over ordinary least squares (OLS) regression.

To address RQ1, Model 1 was estimated with the three double-centered predictors as fixed effects. The model fit improved substantially over the null model, Δ*χ*^2^ (12) = 4008.50, *p* < 0.001. The marginal R^2^ was 0.493, indicating that the three predictors explained 49.3% of the variance in romantic judgment. Details are shown in Table 2. All three predictors significantly and positively predicted romantic judgment, supporting H1, H2, and H3 (see Table 2). Attractiveness was the strongest predictor [*b* = 0.535, *SE* = 0.008, *p* < 0.001, 95% CI (0.518, 0.552)], such that a one-unit increase in perceived attractiveness was associated with a 0.535-unit increase in romantic judgment, indicating that H1 was supported. Connectedness also significantly predicted romantic judgment [*b* = 0.299, *SE* = 0.010, *p* < 0.001, 95% CI (0.279, 0.319)], as did autonomy, though with a smaller effect [*b* = 0.130, *SE* = 0.009, *p* < 0.001, 95% CI (0.113, 0.147)]. H2 and H3 were supported. These findings indicate that all three perceptual dimensions independently contribute to romantic judgment.

**Table 2:** Summary of cross-classified mixed effects model (Model 1).

Predictor	*B*	*SE*	*t*	95% CI for *B*	*p*
Intercept	4.824	0.076	63.886	[4.676, 4.972]	< 0.001
Attractiveness	0.535	0.008	63.230	[0.518, 0.552]	< 0.001
Autonomy	0.130	0.009	14.787	[0.113, 0.147]	< 0.001
Connectedness	0.299	0.010	29.068	[0.279, 0.319]	< 0.001

All continuous predictors were double-centered before analysis. CI, confidence interval.

**Figure 3:**
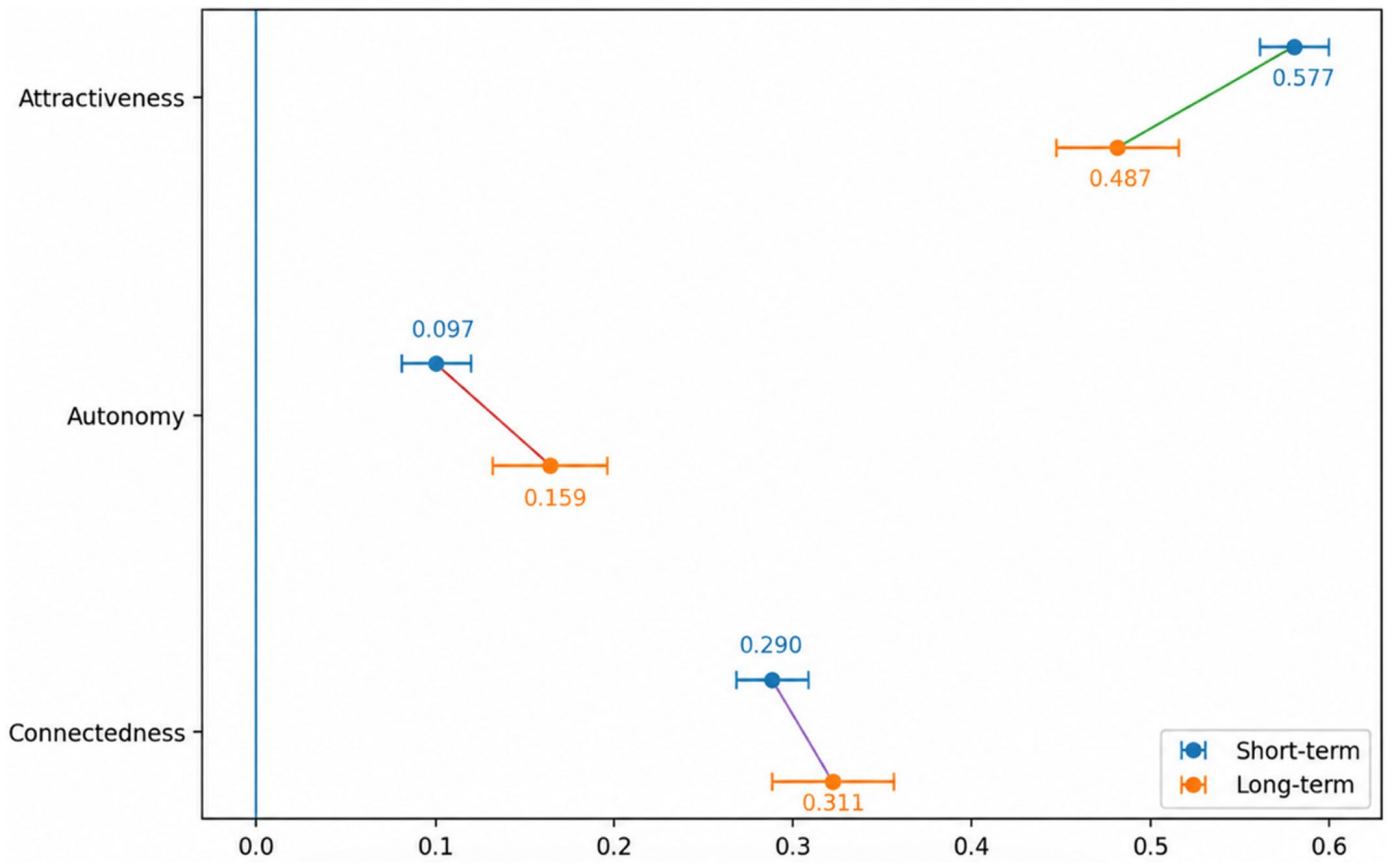
Simple slopes of attractiveness, autonomy, and connectedness predicting romantic judgment across relationship contexts.

To address RQ2, Model 2 added the main effect of relationship context (short-term vs. long-term) and its two-way interactions with each predictor. Model 2 fit significantly better than Model 1, Δχ^2^ (4) = 53.23, *p* < 0.001, indicating that the interactions collectively improved model fit. The main effect of context (long-term) was significant [*b* = 0.135, *SE* = 0.034, *p* < 0.001, 95% CI (0.067, 0.202)], indicating that overall romantic judgment was higher in the long-term than the short-term context when predictors were held at their means. Details are shown in Table 3. The Attractiveness × Context interaction was significant and negative [*b* = −0.090, *SE* = 0.017, *p* < 0.001, 95% CI (−0.122, −0.058)], indicating that the effect of attractiveness on romantic judgment was weaker in the long-term context. Simple slopes confirmed this pattern: attractiveness positively predicted romantic judgment in both the short-term context (*b* = 0.577, *p* < 0.001) and the long-term context (*b* = 0.487, *p* < 0.001), but the effect was stronger in the short-term context. Thus, H4 was supported. Simple slopes are presented in Figure 3. The Autonomy × Context interaction was significant and positive [*b* = 0.062, *SE* = 0.018, *p* < 0.001, 95% CI (0.027, 0.097)]. Contrary to H5, this interaction indicated that the effect of autonomy was stronger in the long-term context than in the short-term context. Simple slopes showed that autonomy positively predicted romantic judgment in both the short-term context (*b* = 0.097, *p* < 0.001) and the long-term context (*b* = 0.159, *p* < 0.001), but its effect was stronger in the long-term context. Therefore, H5 was not supported. The Connectedness × Context interaction was non-significant [*b* = 0.021, *SE* = 0.021, *p* = 0.310, 95% CI (−0.019, 0.061)], indicating that the effect of connectedness on romantic judgment did not differ reliably between short-term and long-term contexts. Simple slopes showed that connectedness positively predicted romantic judgment in both the short-term context (*b* = 0.290, *p* < 0.001) and the long-term context (*b* = 0.311, *p* < 0.001), but the difference was not statistically reliable. Therefore, H6 was not supported: although connectedness showed a slightly stronger effect in the long-term context, the interaction did not reach statistical significance.

**Table 3:** Summary of cross-classified mixed effects model (Model 2).

Predictor	*B*	*SE*	*t*	95% CI for *B*	*p*
Intercept	4.757	0.077	62.092	[4.607, 4.907]	< 0.001
Attractiveness	0.577	0.011	51.548	[0.555, 0.599]	< 0.001
Autonomy	0.097	0.013	7.464	[0.072, 0.123]	< 0.001
Connectedness	0.290	0.015	19.454	[0.261, 0.319]	< 0.001
Context (long-term)	0.135	0.034	3.924	[0.067, 0.202]	< 0.001
Attractiveness × context	−0.090	0.017	−5.436	[−0.122, −0.058]	< 0.001
Autonomy × context	0.062	0.018	3.471	[0.027, 0.097]	< 0.001
Connectedness × context	0.021	0.021	1.017	[−0.019, 0.061]	0.310

All continuous predictors were double-centered before analysis. Context was dummy-coded (0 = short-term, 1 = long-term). CI, confidence interval.

Taken together, perceived attractiveness, autonomy, and connectedness each independently predicted romantic judgment, with attractiveness emerging as the dominant predictor. H1, H2, and H3 were supported. The influence of attractiveness and autonomy was significantly moderated by relationship context. Attractiveness exerted a stronger effect in short-term judgment, and autonomy unexpectedly exerted a stronger effect in long-term rather than short-term judgment. Although connectedness showed a slightly stronger effect in the long-term context as predicted, the interaction did not reach statistical significance. H4 was supported; H5 and H6 were not supported.

## 5 Discussion

The present study tested whether perceived attractiveness, connectedness, and autonomy derived from female dress positively predict men’s romantic partner judgment and whether these associations are moderated by relationship context. The findings provided full support for the main-effect hypotheses: attractiveness, connectedness, and autonomy, each of which positively predicted romantic judgment, supporting H1, H2, and H3. The moderation hypotheses received partial support. As predicted, attractiveness exerted a stronger positive effect in the short-term context than in the long-term context, supporting H4. However, H5 was not supported: contrary to the prediction that autonomy would have a stronger effect in short-term judgment, autonomy showed a stronger positive effect in the long-term context. H6 was not supported: although connectedness showed a slightly stronger effect in the long-term context as predicted, the interaction did not reach statistical significance. These findings suggest that dress-based cues are not weighed uniformly across mating contexts.

### 5.1 Dress-based perceptual predictors of romantic judgment

Consistent with H1, perceived attractiveness positively predicted romantic judgment and emerged as the strongest predictor among the three perceptual dimensions [*b* = 0.535, *SE* = 0.008, *p* < 0.001, 95% CI (0.518, 0.552)]. This pattern aligns with extensive prior research documenting the central role of attractiveness in romantic evaluations (e.g., Singh, 1993; Thornhill and Gangestad, 1999). Evolutionary perspectives suggest that attractiveness cues are prioritized in mate assessment, a view supported by cross-cultural evidence using facial stimuli (Buss, 1989; Little et al., 2011). Although the present study did not assess facial or bodily features, reproductive value, or genetic quality (e.g., Confer et al., 2010; Currie and Little, 2009; Langlois et al., 2000; Rhodes, 2006), the current findings indicate that attractiveness judgments formed from dress images were highly associated with romantic desirability ratings. This suggests that the dress-based attractiveness may serve as a visual basis for romantic judgments.

A complementary account is provided by the halo effect (Dion et al., 1972), wherein attractive stimuli tend to elicit broader positive evaluations. In the present study, where judgments were based solely on dress images, attractiveness may have served as a primary anchor for romantic assessments under constrained informational conditions (Ambady and Rosenthal, 1992; Hester and Hehman, 2023). Although the present analyses took several steps to reduce the possibility of the halo effect, they remain limited in ruling out a shared global evaluative tendency. Confirmatory factor analysis and double-centering support construct separability and reduce some sources of participant-level and dress-level bias, but they do not fully eliminate the possibility that part of the observed association reflects a shared evaluative process when the same participant rated all constructs for the same stimulus in immediate succession. In addition, the design did not directly measure or control participants’ overall liking of each dress beyond perceived attractiveness. Such liking may have simultaneously contributed to higher ratings of autonomy, connectedness, and romantic judgment. Therefore, the unique effects observed in the present models should be interpreted cautiously as statistically differentiated associations rather than as definitive evidence that each appraisal dimension operated independently. Future research should measure general liking or style preference separately and include it as a control variable.

Beyond the dominant role of attractiveness, connectedness (*b* = 0.299) and autonomy (*b* = 0.130) also positively predicted romantic judgment, consistent with H2 and H3, with connectedness emerging as the stronger of the two secondary predictors. Although their effect sizes were smaller than that of attractiveness, these results suggest that romantic judgment may involve additional dress-based impressions beyond attractiveness alone. The stronger predictive weight of connectedness is broadly consistent with theoretical frameworks emphasizing affiliation, warmth, and getting along as central dimensions of social perception and relationship evaluation (Baumeister and Leary, 1995; Baumeister et al., 2001; Eisenbruch and Krasnow, 2022; Fiske et al., 2007). The weaker yet significant autonomy than connectedness is consistent with prior theory, as the latter may reduce uncertainty when getting along, while the former, though valued, may be less decisive in the absence of the latter (Berghman and Hekkert, 2017; Fiske et al., 2007; Haselton and Nettle, 2006; Hornsey and Jetten, 2004). People must balance the demands of connectedness with autonomy (Anderson et al., 2015; Buss and Schmitt, 2019; Rozin and Royzman, 2001).

This result should be interpreted narrowly in relation to the actual item content and bound by the study’s design. As with any impression-based rating paradigm, the dimensions assessed here reflect participants’ subjective judgments of dress stimuli. Similar to attractiveness, these associations remain bounded by the impression-based and concurrent rating design. These possibilities do not invalidate the present findings, but they do suggest that future research using more targeted or behaviorally grounded measurement approaches would help clarify the specific qualities underlying the observed associations with romantic judgment.

### 5.2 Moderation by relationship contexts

The present study extends beyond documenting the independent contributions of each predictor by examining whether their weights in romantic judgment were moderated by relationship context, specifically whether participants evaluated targets as short-term or long-term partners. The moderation findings provided a more differentiated account. H4 was supported; H5 and H6 were not supported. Consistent with H4, attractiveness exerted a stronger effect in the short-term context, indicating that physical attractiveness was especially influential when men evaluated short-term romantic desirability. Contrary to H5, autonomy showed a stronger effect in the long-term context rather than the short-term context. For H6, the connectedness and context interaction did not reach significance, though the direction was consistent with the prediction. Before turning to the interactions, the main effect of context is first examined; long-term is significantly higher than short-term (*b* = 0.135, *p* < 0.001). This main effect may reflect that the long-term (vs. short-term) framing has activated a more comprehensive and socially normative evaluative mindset that emphasizes overall partner suitability rather than attractiveness alone (Buss and Schmitt, 1993; Fletcher et al., 1999). Consistent with this interpretation, the everyday dress images used in the present study, lacking overt sexualization, may have better aligned with the long-term mindset.

The significant negative interaction between attractiveness and relationship context (*b* = −0.090, *p* < 0.001) indicates that the predictive weight of physical attractiveness was attenuated in the long-term relative to the short-term evaluation context, supporting H4. While the effect size is small, it nonetheless captures a statistically reliable and theoretically meaningful shift in how attractiveness is weighted across relationship contexts. This pattern is consistent with theoretical accounts suggesting that mate preferences shift according to the evaluative demands of the relational goal the perceiver holds in mind (Buss and Schmitt, 1993; Confer et al., 2010; Gangestad and Simpson, 2000). In short-term contexts, visible attractiveness cues may be weighted as more immediately relevant indicators of mate value, whereas in long-term contexts, perceivers appear to integrate a broader range of cues, reducing the relative dominance of attractiveness alone. This recalibration is theoretically expected and replicates patterns documented in the broader mate preference literature.

The positive interaction between autonomy and relationship context (*b* = 0.062, *p* < 0.001) represents the most nuanced finding of the present study. Contrary to H5, autonomy emerged as a stronger predictor of romantic desirability in the long-term context than in the short-term context. H5 was derived from prior literature suggesting that autonomy cues are less favored in long-term relational contexts where compatibility and relational investment are primary criteria (e.g., Buss and Schmitt, 1993; Kenrick et al., 1990; Li and Kenrick, 2006). The unsupported H5 should be interpreted in relation to how autonomy was operationalized in the present study. The scale focuses on observers’ interpretations of what autonomous self-presentation communicates in a mating context; however, it does not directly assess behavioral independence, actual self-sufficiency, or stable personality traits. While this approach captures autonomy perceived in everyday appearance cues, there are some differences from established behavioral direct autonomy measurement. Therefore, the present finding runs counter to this expectation, and its interpretation warrants caution.

An additional possibility is that the three autonomy items activated overlapping yet conceptually distinct inferences. Although the scale was intended to capture perceived autonomy, each item also evokes related constructs such as personal independence (Fiske et al., 2007; Hester and Hehman, 2023). Rather than signaling nonconformity or relational disinterest, dress-based autonomy cues may have evoked a more favorable composite evaluation that combined impressions of independence, self-expression, uniqueness, and individuality, any of which could independently elevate romantic judgment in the long-term context (Naumann et al., 2009; Shackelford et al., 2005). This inferential overlap may explain why autonomy predicted greater long-term desirability. As autonomy was operationalized through dress-based impressions rather than direct behavioral or self-report measures, the present data cannot adjudicate among these possibilities, and the concurrent rating design precludes causal inference about which inferred qualities drove the observed association. The unexpected direction of this effect, therefore, remains an open empirical question that future research with more targeted measurement approaches would be better positioned to address, such as employing multi-dimensional autonomy scales that disentangle agentic self-direction, expressive nonconformity, and relational autonomy.

The non-significant trend for connectedness in the predicted direction (*b* = 0.021, *p* = 0.310), combined with the significant autonomy interaction, suggests that long-term desirability may depend on a combination of relational connection and individual agency rather than either dimension alone. Autonomy is not necessarily opposed to long-term relational investment. Instead, commitment from a partner who appears self-directed and autonomous may be perceived as more intentional and psychologically stable (Knee et al., 2013; Kluwer et al., 2020). This points to a more complex picture of long-term cue integration than the original theoretical framework anticipated and highlights the value of examining these dimensions jointly rather than in isolation.

Taken together, the three interaction effects reveal a coherent pattern of context-sensitive cue weighting in romantic judgment. Attractiveness anchored judgment most strongly in the short-term context, supporting H4. Connectedness showed a trend in the predicted long-term direction but did not reach significance, leaving H6 unsupported. Autonomy showed the opposite of the predicted pattern, failing to support H5, and suggesting that autonomy-signaling dress may acquire long-term relevance when interpreted as a cue of competence and intentional commitment. These findings refine the initial theoretical framework by showing that dress-based cues differ in their relational meaning across mating contexts. Critically, the present findings demonstrate that this contextual sensitivity extends to dress-based impression formation, where relevant cues are inferred from a single static visual stimulus, suggesting that the weighting of dress-based cues in romantic judgment varies across evaluative frames.

## 6 Conclusion

The present study examined how dress-based perceptions of attractiveness, autonomy, and connectedness relate to men’s judgments of romantic partners across short- and long-term relationship contexts. Extending beyond face-based paradigms, the findings demonstrate that clothing serves as a medium through which observers perceive not only physical attractiveness but also interpersonal dispositions relevant to romantic judgments. While attractiveness dominated these judgments, particularly in short-term contexts, both connectedness and autonomy emerged as significant secondary predictors, underscoring that romantic judgment integrates multiple dimensions of social perception. Notably, the moderating role of relationship context revealed a nuanced pattern: although autonomy was hypothesized to be less favorable for long-term mating, it served as a stronger predictor in this context. These findings suggest that dress-based autonomy cues may reflect perceived competence and self-sufficiency rather than mere nonconformity, potentially explaining their greater evaluative weight in long-term contexts. These results refine existing mate-preference frameworks by illustrating how the functional meaning of appearance cues shifts with relational goals, highlighting the complexity of impression formation in romantic judgments.

Several limitations should be noted. First, all predictors and the outcome were measured concurrently from the same dress stimuli, meaning that the observed associations reflect covariation among ratings elicited by the same stimulus rather than established causal antecedents and consequences. Future research employing experimental manipulation of specific dress features would be better positioned to establish directional effects. Second, because attractiveness, autonomy, connectedness, and romantic judgment were all rated in immediate succession for each stimulus, the observed associations may partly reflect shared evaluative tendencies arising from a common impression formation process rather than the independent influence of separable cues. Double-centering reduced interfering factors, and the confirmatory factor analysis supported the discriminant validity of the three predictors, providing some analytic mitigation of this concern. However, it should be noted that the analyses do not fully eliminate the possibility that part of the observed association reflects a shared evaluative process when the same participant rated all constructs for the same stimulus in immediate succession. In addition, the design did not directly measure or control participants’ overall liking of each dress beyond perceived attractiveness. Such liking may have simultaneously contributed to higher ratings of autonomy, connectedness, and romantic judgment. Future research using sequential or temporally separated rating procedures, or designs in which cue dimensions are experimentally varied independently, would more directly address this limitation.

Third, the use of condition-specific romantic judgment items and an embedded attention check item provided some assurance of task engagement, but no dedicated manipulation check for the relationship context was administered. Future research should include an explicit manipulation check to verify that the context induction operated as intended. Fourth, the study relied on ten digitally modelled dress images, and the restricted stimulus set may have limited statistical power to detect moderation effects, as suggested by the non-significant connectedness interaction. The exclusive use of male perceivers and female dress stimuli also limits generalizability; whether comparable patterns emerge in female perceivers or same-sex evaluation remains an open question. Fifth, the sample represents a relatively specific cultural context, and the extent to which these findings generalize across cultures warrants investigation, given that dress norms and their associated social meanings vary considerably across societies. Future research would benefit from a broader and more varied stimulus set, inclusion of female perceivers, manipulation checks for relational context, and cross-cultural replication.

Overall, the pattern of supported and unsupported hypotheses suggests that beautification is not a single attractiveness signal but a multidimensional cue system whose associations with romantic judgment shift across relationship contexts. These findings move the field beyond an attractiveness-centered account of female beautification and toward a more complete understanding of the social-perceptual processes through which dress-based cues relate to male romantic evaluation.

## Data Availability

The datasets presented in this study can be found in online repositories. The names of the repository/repositories and accession number(s) can be found in the article/supplementary material.
